# Applying machine learning and predictive modeling to retention and viral suppression in South African HIV treatment cohorts

**DOI:** 10.1038/s41598-022-16062-0

**Published:** 2022-07-26

**Authors:** Mhairi Maskew, Kieran Sharpey-Schafer, Lucien De Voux, Thomas Crompton, Jacob Bor, Marcus Rennick, Admire Chirowodza, Jacqui Miot, Seithati Molefi, Chuka Onaga, Pappie Majuba, Ian Sanne, Pedro Pisa

**Affiliations:** 1 Health Economics and Epidemiology Research Office, Department of Internal Medicine, School of Clinical Medicine, Faculty of Health Sciences, University of the Witwatersrand, 39 Empire Road, Parktown, Johannesburg, South Africa; 2 Palindrome Data, Cape Town, South Africa; 3 Right to Care, Johannesburg, South Africa; 4 Department of Global Health, Boston University School of Public Health, Boston University, Boston, USA; 5 Department of Epidemiology, Boston University School of Public Health, Boston University, Boston, USA; 6 Department of Human Nutrition and Dietetics, Faculty of Health Sciences, University of Pretoria, Pretoria, South Africa

**Keywords:** Epidemiology, Outcomes research, Computational science

## Abstract

HIV treatment programs face challenges in identifying patients at risk for loss-to-follow-up and uncontrolled viremia. We applied predictive machine learning algorithms to anonymised, patient-level HIV programmatic data from two districts in South Africa, 2016–2018. We developed patient risk scores for two outcomes: (1) visit attendance ≤ 28 days of the next scheduled clinic visit and (2) suppression of the next HIV viral load (VL). Demographic, clinical, behavioral and laboratory data were investigated in multiple models as predictor variables of attending the next scheduled visit and VL results at the next test. Three classification algorithms (logistical regression, random forest and AdaBoost) were evaluated for building predictive models. Data were randomly sampled on a 70/30 split into a training and test set. The training set included a balanced set of positive and negative examples from which the classification algorithm could learn. The predictor variable data from the unseen test set were given to the model, and each predicted outcome was scored against known outcomes. Finally, we estimated performance metrics for each model in terms of sensitivity, specificity, positive and negative predictive value and area under the curve (AUC). In total, 445,636 patients were included in the retention model and 363,977 in the VL model. The predictive metric (AUC) ranged from 0.69 for attendance at the next scheduled visit to 0.76 for VL suppression, suggesting that the model correctly classified whether a scheduled visit would be attended in 2 of 3 patients and whether the VL result at the next test would be suppressed in approximately 3 of 4 patients. Variables that were important predictors of both outcomes included prior late visits, number of prior VL tests, time since their last visit, number of visits on their current regimen, age, and treatment duration. For retention, the number of visits at the current facility and the details of the next appointment date were also predictors, while for VL suppression, other predictors included the range of the previous VL value. Machine learning can identify HIV patients at risk for disengagement and unsuppressed VL. Predictive modeling can improve the targeting of interventions through differentiated models of care before patients disengage from treatment programmes, increasing cost-effectiveness and improving patient outcomes.

## Introduction

Globally, it is estimated that in 2019, there were more than 38 million people living with HIV (PLHIV), of which 8 million (20%) were living in South Africa^1^. Despite wide-scale HIV prevention efforts and the adoption of a ‘treat all’ strategy^2^, where treatment also provides a prevention benefit, South Africa still saw 230,000 new infections in 2020 alone^3^. While the implementation of the treat-all policy has increased the uptake of antiretroviral therapy (ART) with recent estimates indicating ART coverage levels of approximately 70%^4^, the challenge of patient retention within public sector ART programs in South Africa remains of concern^5^. In 2020, only 66% of PLWH in South Africa were virally suppressed^3^. To optimize South Africa’s HIV response and reach targets of 95% of PLHIV tested, 95% of those on ART and 95% of ART patients virally suppressed, the numbers of patients initiating and successfully maintaining viral suppression on antiretroviral therapy must increase^6,7^.

Current interventions to address disengagement from care are typically reactive in nature; attempting to identify, trace and return to care those who have already disengaged from treatment programs^8–10^. These approaches are costly in terms of human capital and do not optimize resource allocation according to risk prioritization and are thus not universally provided. If we could more accurately identify patient groups at risk of disengagement and how to target available interventions to these groups while still engaged in care, resources could be allocated more efficiently and precisely, addressing higher risk groups while simultaneously reducing health care worker burden, a major driver of cost in health care.

Borrowing from techniques widely utilized in credit scoring^11^, we aimed to determine whether machine learning applied to routinely collected longitudinal HIV phenotypic and clinical outcome data in South African programmes could consistently identify patients at risk of poor outcomes in terms of two key programmatic outcomes: (1) attendance at next scheduled clinic visit and (2) suppression of next HIV viral load (VL).

## Methods

### Data source and study population

**Figure 1:**
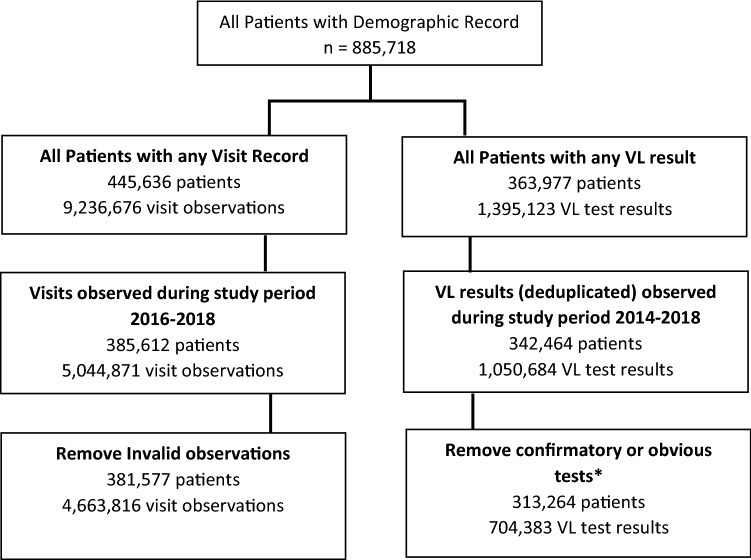
Flow chart of data source inclusion. *Notes: All VL tests immediately following a high test (> = 1000) and subsequent tests done < 6 months after first test removed. All visits with a ‘next appointment date’ that was either missing or more than 99 days were removed.

We utilized routinely collected, anonymised, longitudinal data from patients accessing HIV care and treatment at public sector treatment sites in Mpumalanga and the Free State between January 2016 and December 2018. Data were used to train two predictive models: (1) retention model and (2) VL suppression model. Data included demographic, clinical and antiretroviral treatment records indicating clinical visits, laboratory test results and other key HIV indicators. For this study, we included records from patients who had accessed HIV care (observed in routine HIV electronic records) and, during the period 2016–2018, had at least one recorded clinic visit (Fig. 1).

### Outcome variables

We defined two *primary outcomes* for this analysis: one for each model. First, for the retention model, we defined retention based on the 2019 PEPFAR definitions of loss to follow-up according to visit attendance^12^. A visit was defined as “attended” if a clinic visit was observed before or within 28 days of the next scheduled appointment date. Patients with an observed attended visit were considered retained in care at that time. A visit was considered “missed” if a patient did not attend within 28 days of their scheduled appointment, and at that point, the patient was considered not retained in care. Second, for the VL suppression model, a patient was considered to have an unsuppressed VL if the VL test result indicated an HIV viral load of > 400 copies/mL. During the study period, national treatment guidelines indicated VL monitoring for all patients on ART with the first VL at six months after initiation and then yearly thereafter^13^. These two primary outcomes were targeted for model building, as both represent established clinical treatment outcomes and are objectively defined (diagnostically measurable reading). Each visit and VL test were considered unique events, such that a single patient would have each visit and VL test outcome measured and a patient’s outcomes (missed/attended and suppressed/unsuppressed) could change across subsequent visits and tests.

For the VL model, we included patient records with at least one VL test recorded between 2016 and 2018, while for the retention model, visit data were included only where a valid next scheduled visit date was recorded (between 14 and 99 days after the current visit). Patients without any recorded visit to a recognized three-drug highly active antiretroviral treatment regimen were excluded from analysis. For both models, we included patients commencing ART prior to 2016 provided that we observed visit or viral load data for that patient during the period 2016–2018.

In addition, for the VL outcome, we excluded any VL tests from analysis that followed a high VL value (> 1000 copies/mL) due to the majority of these being repeat confirmatory tests and thus having a high probability of also being unsuppressed. We also removed any VL observation that had a recent prior VL (within the previous 6 months) again due to the high probability of concordance between these results (Fig. 1). These exclusions remove results that are more clinically “obvious” to a human observer and easy for the algorithm to correctly classify. This allowed us to build a model focusing on patients who have either previously been virologically stable or not yet been tested, i.e., have the prediction focus on those most at risk of converting to the new ‘state’ of virologically unsuppressed.

### Predictor variables

All raw data available in the source datasets were considered potential predictors of retention and VL suppression for the model building process. The large number of potential predictor variables, 75, were reduced to a prioritised set of predictor input features for each model, through a preliminary process of recursive feature selection and elimination against predictive metrics, producing the most predictive set of input features (Table 1A, B). These included data characterizing patient demographics (e.g., age, gender), drug regimen data, visit history and patterns (e.g., number of visits, frequency of missed visits, number of treatment facilities attended), ART treatment history (e.g., current regimen, number of regimen switches) and ART monitoring laboratory test results. Additional potential predictor variables were calculated or inferred from existing data. For example, the date of each visit was used to create variables indicating the day of the week or day of the month that the visit was scheduled for.

**Table 1:** Input features for the (A) retention model and (B) unsuppressed VL model.

Input feature	Description
**(A) Retention model**	
3 days late ratio	Ratio of patient’s historical visits which they have been late by 3 + days
28 days late count	How many times previously has the patient been late by 28 + days
Visits at this facility	How many visits has the patient had at this facility (in total)
Number of VL tests	The number of VL tests the patient has had (in total)
Months since first visit	Months since the patients’ earliest recorded visit in patient record
Months since last visit	Months since the patients’ most recent recorded visit in patient record
Current age	Patient’s age at the current visit
Day of month next appointment	Day of the month (1–31) that the next appointment is schedule for
Last VL value	Patient’s last VL test value in copies/mL
Day of week next appointment	Day of the week (1–7) that the next appointment is schedule for
# Visits on current regimen	The number of recorded sequential visits the patient has had on the current ART treatment regimen
Is male	Gender of the patient encoded as 1 for Male, and 0 for Female
#Missed Months	The number of times the patient has had a whole month with no recorded clinical visit
**(B) Unsuppressed VL model**	
Age started ART	Patient’s age on starting ART
Last VL value	Patient’s last VL test value in copies/mL
Duration on ART	Total number of months on ART treatment ever
Month of test	Calendar Month of Test
#Visits on current regimen	The number of recorded sequential visits the patient has had on the current ART treatment regimen
# of visits ever	The number of recorded visits the patient has had ever
Visits miss ratio	The proportion of patient’s historical visits which they have missed completely
Months since last VL test	Months since a last VL test was taken
Months since last visit	Months since the patient was last recorded to have visited a facility
Year of test	The year of the test
# Missed visits ever	The number of appointments the patient has missed ever

### Model selection criteria

Three classification algorithms were tested, namely, logistic regression, random forest and AdaBoost. Logistic regression is an example of a linear classifier and is best suited for separating the predictor hyperspace with a linear boundary between the two classes. The random forest approach is an ensemble approach consisting of a collection of different randomly composed decision trees whose results are aggregated (through a tournament style voting process) into one best breed result. Their random nature often limits overfitting while controlling error, making them attractive modeling tools for complex hyperspaces with nonlinear separations in classes. Like random forest, AdaBoost (“Adaptive Boosting”) classifier is a meta-estimator that fits a classifier on the original dataset and then fits additional copies of the same classifier on the same dataset; in this case with decision trees as the classifier. However, it uses boosting to evaluate the performance of subtrees sequentially as the training develops instead of waiting to the end as per the random forest method. These three algorithms were selected to test the diverse approaches to learning the input data space that each algorithm represents and to evaluate the fit of each algorithm to the study dataset with respect to the different strengths, weaknesses and applications of each.

Both ensemble methods are designed to predict a binary outcomes based on various inputs but do not have the linear separation limitation of logistic regression. Both ensemble learning algorithms have the benefit of upweighting the combination of variables at certain thresholds on how they correlate with an output variable—thus often being more sensitive to minority classes or multiple subgroups that may emerge in the data.

### Model building

In total, 75 predictor variables (raw and engineered) were investigated for inclusion in the models. Laboratory, visit and patient demographic data were linked and deduplicated, and invalid or implausible fields were removed, as outlined in Fig. 1. This analytic set was evaluated to establish the baseline prevalence of each outcome (% of all visits that were late by > 28 days and % of all VL tests that were unsuppressed). The analytic set was then randomly sampled on a 70/30 split into a training (70%) and test (30%) set. The training set for each outcome was then downsampled to equal shares (50% each) of positives (visits classified as missed in the retention dataset and unsuppressed VL result in the VL dataset) and negatives (visits classified as attended and suppressed VL result) to produce a balanced training set of positive and negative examples for the classification algorithm to learn from. This step also addresses bias tendency toward predicting the majority class observed in many machine learning algorithms known as the *class imbalance problem*^14^ and as such should be interpreted as the primary analytic output. An additional model was tested with unbalanced class membership (60–40) in the training data; we present the results of these for comparison and demonstration of this principle.

The classifier algorithm was trained using the training data set, consisting of input of predictor variables as well as the specified target outcomes per visit. This training produces an optimal configuration (predictive model) such that the predictor variables correspond to the specified target outcomes as often as possible. After the model was trained, we separated the unseen test set into predictor variable data and outcome data. The model then was tested on the unseen data set by generating predicted outcomes using the predictor variables from the unseen test set as input for each observation. In this case, observations were each scheduled visit for the retention outcome and each viral load test for the VL outcome. The predictions were then scored for accuracy against the known outcomes in the unseen test set (whether a scheduled visit was attended within 28 days for the retention model and whether a viral load test result was suppressed for the VL suppression model).

### Model evaluation and risk grouping

The model’s performance was evaluated in terms of accuracy (proportion of observations correctly classified by the algorithm among all observations in the unseen test set), sensitivity (the proportion of known positive outcomes in the unseen test set that are correctly identified as such by the algorithm), positive predictive value also known as precision (the proportion of positive outcomes predicted by the algorithm that correspond to known positive outcomes in the unseen test set) and specificity (the proportion of known negative outcomes in the unseen test set that are correctly identified as such). Next, we utilized the area under the curve (AUC) of a receiver operating characteristic (ROC) curve to evaluate the broad predictive classification performance of the model. A range of 0.5 indicates no predictive power, while 1.0 indicates perfect predictive power.

Finally, we repeated the fitting of the model after looking individually at the highest- and lowest-ranked patients and each of their characteristics. We manually checked for areas of obvious commonality and where the algorithm might be choosing obvious answers or making erroneous assumptions.

### Ethics approval

No experiments on animal or human tissues were conducted as part of this study. All methods relating to the analysis of de-identified data from human subjects were approved by and carried out in accordance with relevant guidelines and regulations as set out by the Human Research Ethics Committee of the University of the Witwatersrand (Medical). This study involved secondary analysis of deidentified data collected as part of routine care, and the requirement for individual patient consent was waived by the Human Research Ethics Committee of the University of the Witwatersrand for protocols M140201 and M210472 during the study approval.

## Results

In total, 445,636 patients were included in the retention model and 363,977 in the VL model. Nearly one-third (30%) of patients were male, with a median age of 39 years (IQR 31–49 years) at the time of visit. In the retention dataset, patients had a median of 18 (IQR 10–25) visits since entering care and had been in care for a median of 31 (IQR 15–43) months. The vast majority (91%) of patients visited a single facility during the period under analysis.

### Predictor variables and baselines

We generated 75 potential predictor variables per visit and 42 predictor variables per VL test. The retention and VL suppression models were built using the AdaBoost and random forest^15^ binary classification algorithms, respectively, from the scikit-learn^16^ open source project and tested against unseen data to evaluate predictive performance.

For the retention model, the test set consisted of 1,399,145 unseen visits randomly selected from across 2016–2018. The test set’s baseline prevalence of missed visits was 10.5% (n = 146,881 visits), consistent with the LTFU prevalence observed in both the full data set and the training set. This observed baseline was comparable with meta-studies of LTFU at 1 year in South Africa 2011–2015^17^. For the VL suppression model, the dataset was split into training and testing sets, with the test set consisting of 30% (n = 211,315) of the original unseen tests randomly selected from across the study period. In the VL test set, there were 21,679 unsuppressed (> 400 copies/mL) viral load results for a baseline prevalence of unsuppressed VL results of 10.3%.

### Retention model results

We selected two approaches to the training sets: first, the sample was balanced in terms of the output classes (50% missed and 50% not missed visits); and second, with an unbalanced sample—60% not missed and 40% missed visits). The AdaBoost classifier was trained with a 50:50 balanced sample of the modeling set, which resulted in 343,078 of each visit classification (*missed* or *not missed* visits) in the training set. Using the test set, the retention model correctly classified 926,814 of the test set (~ 1.4 m visits) correctly, yielding an accuracy of 66.2% (Table 2A). In total, 89,140 patients missed their scheduled visit and were correctly identified out of a possible 146,881 available known missed visits, yielding a sensitivity of 60.6% for all positives. Conversely, 837,674 visits were correctly identified as not missed out of a total of 1,252,264 visits observed as not missed for a specificity of 67% and a negative predictive value of 94%.

**Table 2:** Late visit model metrics based on (A) balanced and (B) unbalanced training sets.

	Missed visit observed	Missed visit not observed	Total observations	Se/Sp metrics	F1-score
**A: Balanced 50:50 model performance using test set**					
Missed visit predicted	89,140	414,590	503,730	Se = 61%	0.27
Missed visit not predicted	57,741	837,674	895,415	Sp = 67%	0.78
	146,881	1,252,264	1,399,145		
PPV	18%				
NPV	94%				
AUC	0.688				
Accuracy	66.2%				
**B: Unbalanced 60:40 model performance using test set**					
Missed visit predicted	59,739	211,764	271,503	Se = 41%	0.29
Missed visit not predicted	87,040	1,040,602	1,127,642	Sp = 83%	0.87
	146,779	1,252,366	1,399,145		
PPV	22%				
NPV	92%				
AUC	0.688				
Accuracy	78.6%				

The F1 score is the harmonic mean of sensitivity and specificity such that 1.0 is the best score.

Accuracy is the number of correct predictions out of the total number of predictions over the test set observations.

Se = sensitivity; Sp = specificity; PPV = positive predictive value; NPV = negative predictive value.

Next, the AdaBoost classifier was trained with an unbalanced 60:40 sample of the modeling set. This translated into 343,180 missed visits and 514,770 visits attended on time in the training set. The retention model trained on the unbalanced sample correctly classified 1,100,341 of the test set (~ 1.4 m), for an accuracy of 78.6% (Table 2B). However, only 59,739 of the missed visits were correctly identified, yielding a sensitivity of 40.6% for all positives and a false negative rate of 59.3%. The model’s negative predictive value remained high at 92%, further suggesting that attended scheduled visits are easier to identify than missed visits.

**Figure 2:**
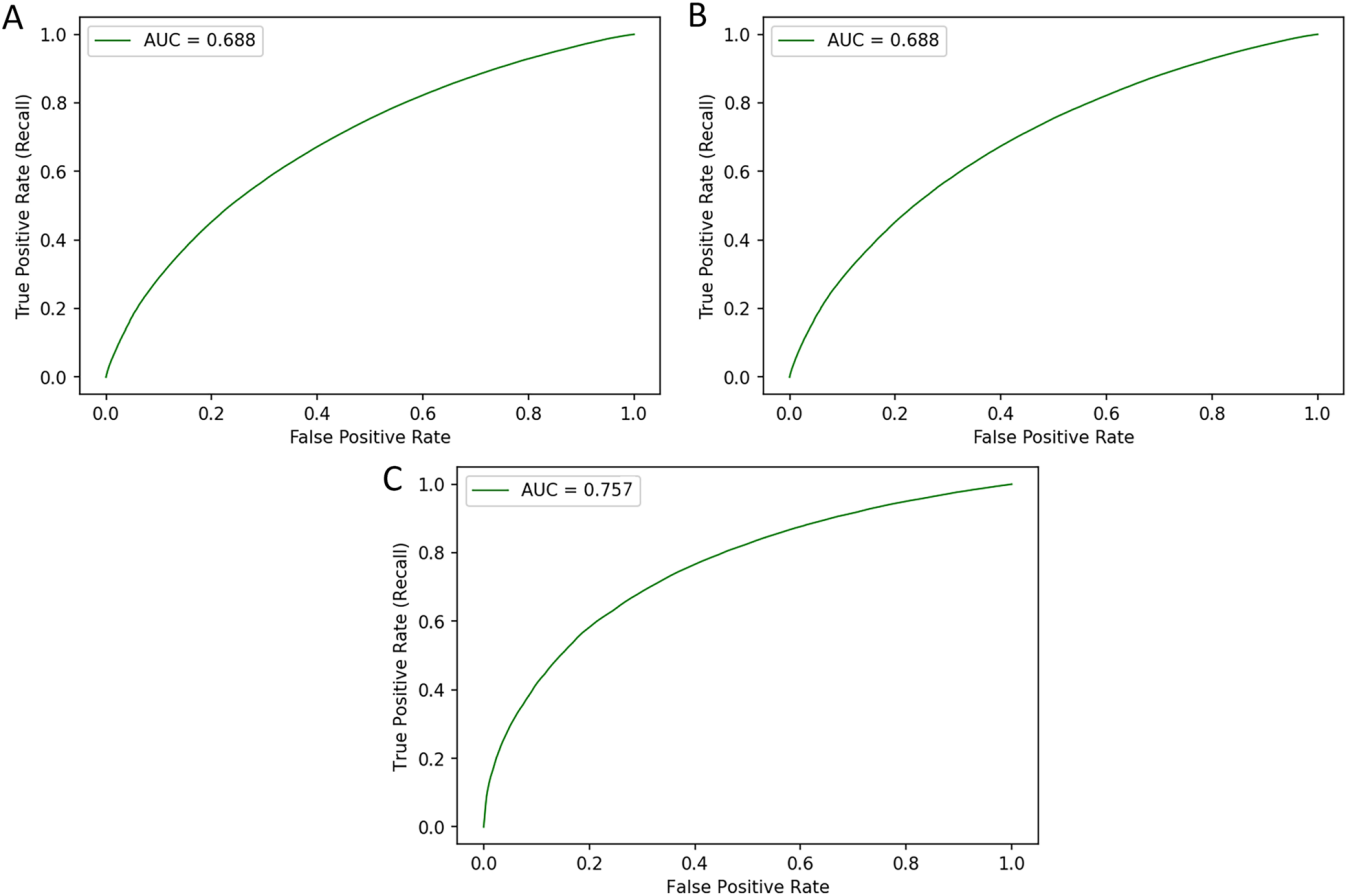
ROC Curve of (**A**) 50:50 balanced late visit classifier, (**B**) 60:40 unbalanced late visit classifier and (**C**) 50:50 balanced unsuppressed VL classifier.

The two models demonstrated the potential trade-off in accuracy, precision and sensitivity that can be manipulated in the training of the models^18^. However, the predictive power or utility of the model to separate between classes—represented by the AUC metric—remained consistent across models. The two ROC curves are depicted in Fig. 2A,B with the same AUC and identical shapes. Whilst this difference of sampling approach demonstrates the manipulation of the metrics, it is important to note that this rebalancing and re-sampling of the training set can also introduce under or misrepresentation of sub classes, with each data set uniquely sensitive to imbalance problems particularly at smaller sample sizes^19,20^.

### Suppressed VL model results

For the suppressed VL model, the final training set was down sampled to 101,976 tests, such that it had a 50:50 balanced sample. The model correctly classified 153,183 VL results out of the test set of 211,315 correctly, yielding an accuracy of 72.5% (Table 3). In total, 14,225 unsuppressed viral load tests were correctly predicted out of a possible 21,679 unsuppressed test results, yielding a sensitivity of 65.6%. The model’s negative predictive value was very high at 95%, again suggesting that suppressed VL results (i.e., lower risk) are simpler to recognize. Overall, the model had an AUC of 0.758 (Table 3, Fig. 2C).

**Table 3:** Unsuppressed VL model metrics based on balanced (50:50) training sets.

	Unsuppressed VL observed	Unsuppressed VL not observed	Total observations	Se/Sp metrics	F1-score
**VL Balanced 50:50 model performance using test set**					
Unsuppressed VL predicted	14,225	50,678	64,903	Se = 66%	0.33
Unsuppressed VL not predicted	7,454	138,958	146,412	Sp = 73%	0.83
	21,679	189,636	211,315		
PPV	22%				
NPV	95%				
AUC	0.758				
Accuracy	72.5%				

The F1 score is the harmonic mean of sensitivity and specificity such that 1.0 is the best score.

Accuracy is the number of correct predictions out of the total number of predictions over the test set observations.

Se = sensitivity; Sp = specificity; PPV = positive predictive value; NPV = negative predictive value.

### Predictor importance

**Figure 3:**
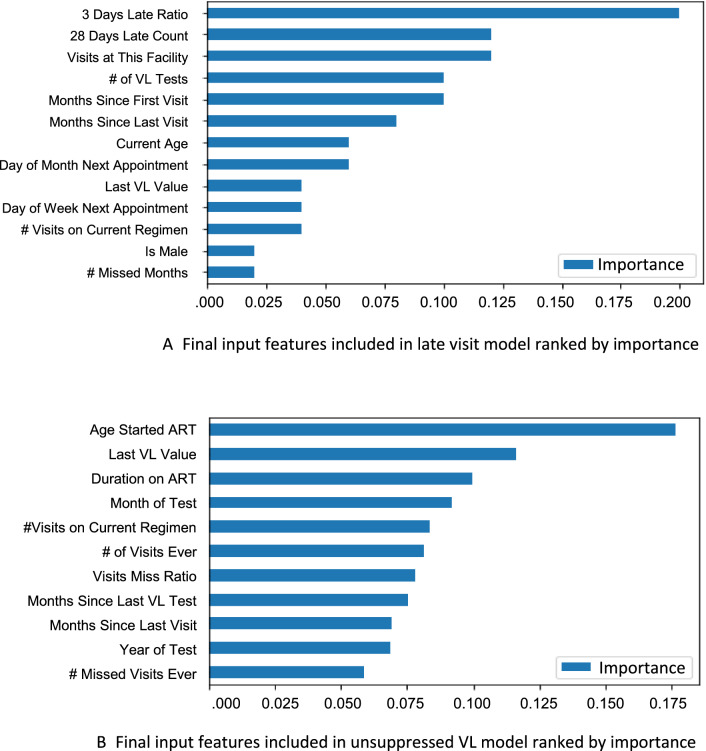
(**A**) Final input features included in late visit model ranked by importance. (**B**) Final input features included in unsuppressed VL model ranked by importance.

The original set of over 75 input predictor variables for the retention model (and 42 for the unsuppressed VL model) were reduced to a more practical number through feature selection using a Random Forest algorithm on all inputs. Random Forest permutes the inputs into trees of different groups of predictors, and the change in predictive power (as measured by AUC) of the model for each permutation was calculated. This process prioritises groups of predictor variables that together improve predictive power and deprioritises those that contribute little or no improvement to AUC. Random Forest was able to rank the relative feature importance of the total input set for each model. Figure 3A,B illustrate their relative importance in helping correctly and repeatedly classify a particular observation as a correct or incorrect prediction of the target outcome. The predictor variables with higher importance help the algorithm distinguish between its classifications more often and more correctly than those with lower importance. For example, in the retention model (Fig. 3A), gender represented in the Boolean variable ‘Is Male’ has some correlation with the missed visit target outcome and measurably more than the eliminated predictor variables that had zero correlation. However, it is clear that the algorithm relied on correlations in the patients’ prior behavior (frequency of lateness, time on treatment, etc.) to segment the risk of outcome, and together, these described more of the difference than gender alone.

Our results indicated that prior patient behavior and treatment history were extremely important in predicting both visit attendance and viral load results in these datasets and that traditional demographic predictor variables were less useful than behavioral indicators. These more powerful predictor variables can also be used to further stratify populations by risk and segment more granularly for targeted interventions and differentiated care.

During feature selection we investigated overfitting to particular features through comparative tests of features permutation importances with the goal of identifying any overfitted but erroneous highly correlated features in the training set that weren’t a reflected phenomenon in the test set (Supplementary Figure 1). We also performed correlation checks on the candidate input features. Rather than assuming that multicollinearity in the input variables was necessarily leading to information loss, during the feature selection phase, we tried several combinations of feature groupings to test the relationship of certain groups against the prediction metrics. The matrix of these feature correlation checks is depicted in Supplementary Figure 2.

We also report the model performance metrics considering various subsets of the ranked input features to determine whether reducing the model to the 10 most important features impacted on performance metrics. As noted in Supplementary table 1, overall model accuracy varied by only 5% comparing a model including only the 5 most important features (62%) with a model including all 75 features (67%). Difference in AUC between these two models was less than 0.04 (Supplementary Figure 3).

## Discussion

In September 2016, the National Department of Health revised its treatment guidelines to extend the availability of ART to all people living with HIV, irrespective of CD4 cell count and stage of disease^2^. This policy, widely referred to as “treat all” or “universal test and treat” (UTT), holds promise to offer substantial advancements not only in the health of those living with HIV^21,22^ but also in the country’s efforts to initiate and retain 95% of all people living with HIV (PLWH) who know their status on antiretroviral therapy (ART) as part of UNAIDS’ global 95-95-95 strategy. However, implementation of a policy such as UTT requires a rapid scale-up and expansion of the ART program on a country-wide level, a shift that is often challenging in communities with the most cases of HIV and the largest ARV programs globally.

As South Africa increases efforts toward 95-95-95 goals, knowing which patients require additional services and interventions to achieve successful treatment outcomes at each step of the care cascade is critical. As national health monitoring systems expand to collect large volumes of increasingly detailed data, the application of data science technology and methodology to these and other data sources holds the potential to improve individual health across population groups. Traditional approaches to estimating the risk of losses from the care cascade and designing interventions to address those losses have not realized the improvements in outcomes they promise. Reasons for disengagement differ widely, yet studies investigating these typically look at individual or combinations of baseline characteristics (usually at a fixed point such as initiation on ART) and then treat large groups (e.g., men or those in specific age ranges) as homogenous. These approaches make two potentially flawed assumptions: (1) it assumes that large subpopulation groups are homogenous and should be addressed as a single entity (for example, adolescent-friendly services), and (2) it assumes predictive characteristics are fixed from a random baseline point of assessment through to specific endpoints ranging anywhere from 6 months to several years after assessment of these features. Behavioral science, however, tells us that behavioral drivers of health seeking patterns not only differ by patient but also over time for individuals.

To address these challenges, we applied machine learning to routinely collected demographic, visit and laboratory data and built a predictive model able to effectively separate high-risk from low-risk patients using a combination of routinely available clinical, laboratory and behavioral (visit patterns) data. With continuous challenges in funding to run and operationalize HIV/TB programmes in South Africa, precision programming pivoted by targeted cost-effective, data driven interventions in an almost near to real-time fashion are critical. This approach is enhanced by the implementation of predictive analytics and machine learning within existing programmes.

Our results suggest some important implications for the application of this methodology in clinical practice. First, utilizing only routinely collected demographic, visit and laboratory data, and depending on the training method, predictive models have the ability to correctly predict attendance in a range of 66–79% of scheduled clinic visits and VL suppression in 76% of viral load test results, two key predictors of UNAID’s 95-95-95 targets. Second, the shape of the ROC curve allows us to identify thresholds and ranges of differing group risk profiles (groups that represent an important share of those with each outcome). For example, in the unsuppressed VL model, 20% of the population accounted for > 50% of the unsuppressed VL results, indicating different risk strata within population groups. Thresholds can be identified to segment the population into risk groups (“Red” = high risk of outcome, “Yellow” = medium risk of outcome and “green” = low risk of outcome). This stratification of risk groups can be leveraged for gains in predictive power estimated through population segments. Targeting at-risk patients before they disengage from care or virally unsuppressed allows for greater resource use efficiencies.

Third, we demonstrated high negative predictive value (94% and 95% for the retention and VL models, respectively) and sensitivity > 60% for both models. This, combined with a low positive predictive value (PPV ~ 20%), potentially indicates that low-risk or so-called “green” patients are readily and accurately identified by the model and have common traits. In contrast, patients at high risk of poor outcomes or so-called “red” patients can be readily identified as “not green” but demonstrated much heterogeneity in terms of presentation and underlying reason for disengaging from care. Or, to paraphrase the Anna Karenina principle^23^, *“all happy patients are alike; each unhappy patient is unhappy in its own way*”. While the models cannot provide the underlying reason for disengagement from care, the ability to identify low-risk patients with high accuracy could prove to be of immense value for health systems, as it allows health care workers to prioritise patients not classified as low risk who may be at higher risk of disengagement, prioritizing time and resources toward these patients for targeted intervention as appropriate to their individual context.

Our results should be interpreted in light of some limitations. First, as anonymised routinely collected facility-level data were used to fit the models, it was not possible to trace missing data or correct erroneously linked visits and laboratory data. Second, facility-level data used to define the outcome in each of the models do not account for all silent transfers to another facility, so to the extent that this outcome misclassification has occurred, we will overestimate missed visits and blur the predictive efforts of the model. Future models would benefit from being fit to national cohorts where a system-wide view accounts for the effect of silent transfers^24^ Third, one of the recognized limitations in the nature of black-box machine learning methods such as classification trees is that while these predictors contribute to differences in risk, we cannot yet fully explain *how* or *why* they contribute within the context of an individual patient. The next step is to understand which values or ranges of the predictor’s spectrum in combination with the other variables at certain levels may correlate with a certain outcome. For example, it is unclear whether the risk relationship between visits and age and poor outcomes is with younger people coming on time or older people being late or vice versa. Additional analysis and modeling activities are underway to provide interpretable descriptions of how the algorithm is able to segment the populations of observations. Finally, much of the understanding as to *why* certain visit or demographic predictors are important may lie in more subtle social nuances related to an individual’s social circumstances and health-seeking behaviors (such as disclosure, employment or family support)^25^. To this end, our approach can be utilized and further refined by applying it to different data sources with richer social and behavioral predictors.

Despite these limitations, the analysis has several strengths. First, our findings highlight the importance of better understanding the risk profile of individuals, supporting recent calls for advancing data science toward precision public health models^26^. Accurately identifying those at risk for poor treatment outcomes will allow for health care services to better triage patients, improving efficiency and resource utilization. By prioritizing those most at-risk, clinics can realize better health outcomes without additional investments in data collection and staff. In addition, the results of the algorithm can also be aggregated and used to risk score population subgroups at the facility level to identify where programs need to target specific interventions.

Second, while most retention interventions are directed at tracing patients disengaged from care and then returning them to care, our model offers the opportunity to shift the focus and resources directed at retention efforts to a period while the at-risk patient is still engaged at the point of care. By anticipating future issues before any visible clinical signs are present (e.g., an unsuppressed VL), clinics can intervene proactively while patients are still accessible, engaged in health services and provide targeted services earlier. Early detection of patients at high risk of becoming virologically unsuppressed has implications not only for the individual patient’s health but also for the risk of onward transmission of the virus and impact on breaking transmission chains^27–29^.

## Conclusions

Predictive models and machine learning can identify and target HIV patients at risk for disengaging from care and not being virally suppressed. Our approach could enable anticipation of future outcomes before any visible signs and/or poor outcomes occur (e.g., an unsuppressed VL) and, most importantly, while the patient is still engaged in care. This affords the opportunity to take a proactive approach to patient management—specific targeted interventions can be designed on identified subsets of the treatment cohorts, allowing for cost-effective differentiated models on care and treatment to be applied across the cascade. This approach could also be extended to other key HIV outcomes, allowing for the use of a cost-effective and precision programming approach.

## Supplementary Information

Supplementary Information.

## Data Availability

The data that support the findings of this study are owned by the South African Government and were used under license for the current study. Access to these is subject to restrictions owing to privacy and ethics policies set by the South African Government, so they are not publicly available. Requests to access these should be directed to pedro.pisa@righttocare.org.

## Abbreviations

ART: Antiretroviral therapy
AUC: Area under the curve
PLHIV: People living with HIV
ROC: Receiver operating characteristic
UTT: Universal test and treat
VL: Viral load
