# Supplementary material for: Applying machine learning and predictive modeling to retention and viral suppression in South African HIV treatment cohorts
Source: Sci Rep. 2022 Jul 26;12:12715. doi: 10.1038/s41598-022-16062-0 (PMC9325703; doi:10.1038/s41598-022-16062-0)
Supplement: Supplementary file 1 — Supplementary Information. [file 41598_2022_16062_MOESM1_ESM.docx]

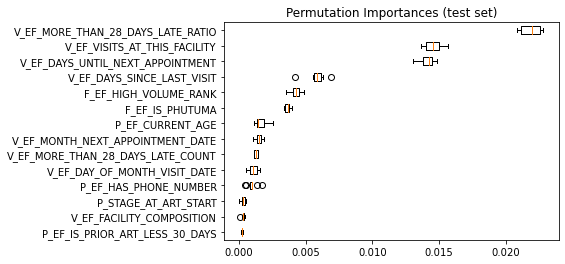

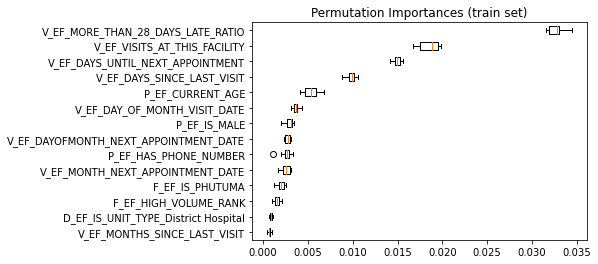


**B**

**A**

**Supplementary Figure 1: Feature permutation importance stratified by training set (A) and test set (B)**


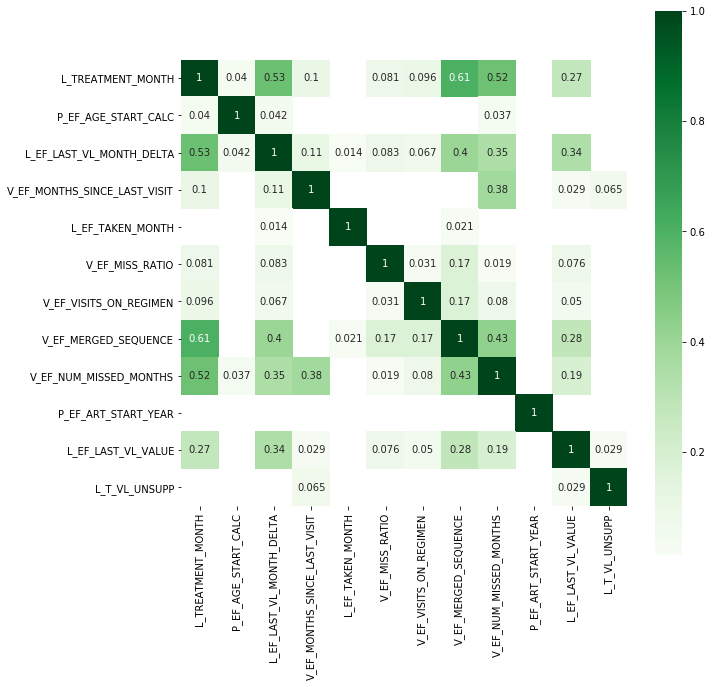


**Supplementary Figure 2: Feature correlation matrix (VL model)**

**Supplementary Table 1: Model performance metrics stratified by number of included features**

| N Features | N | Train % | Test % | Precision | Recall | F1 score | Acc % | AUC |
| --- | --- | --- | --- | --- | --- | --- | --- | --- |
| Top 5 | 711737 | 50% | 10.70% | 0.14 | 0.51 | 0.22 | 62% | 0.603 |
| Top 7 | 436910 | 50% | 9.73% | 0.14 | 0.51 | 0.22 | 64% | 0.612 |
| Top 10 | 711737 | 50% | 10.60% | 0.17 | 0.59 | 0.27 | 66% | 0.674 |
| Top 25 | 436910 | 50% | 9.80% | 0.16 | 0.59 | 0.25 | 66% | 0.674 |
| All 75 inputs | 711737 | 50% | 10.60% | 0.16 | 0.51 | 0.25 | 67% | 0.638 |


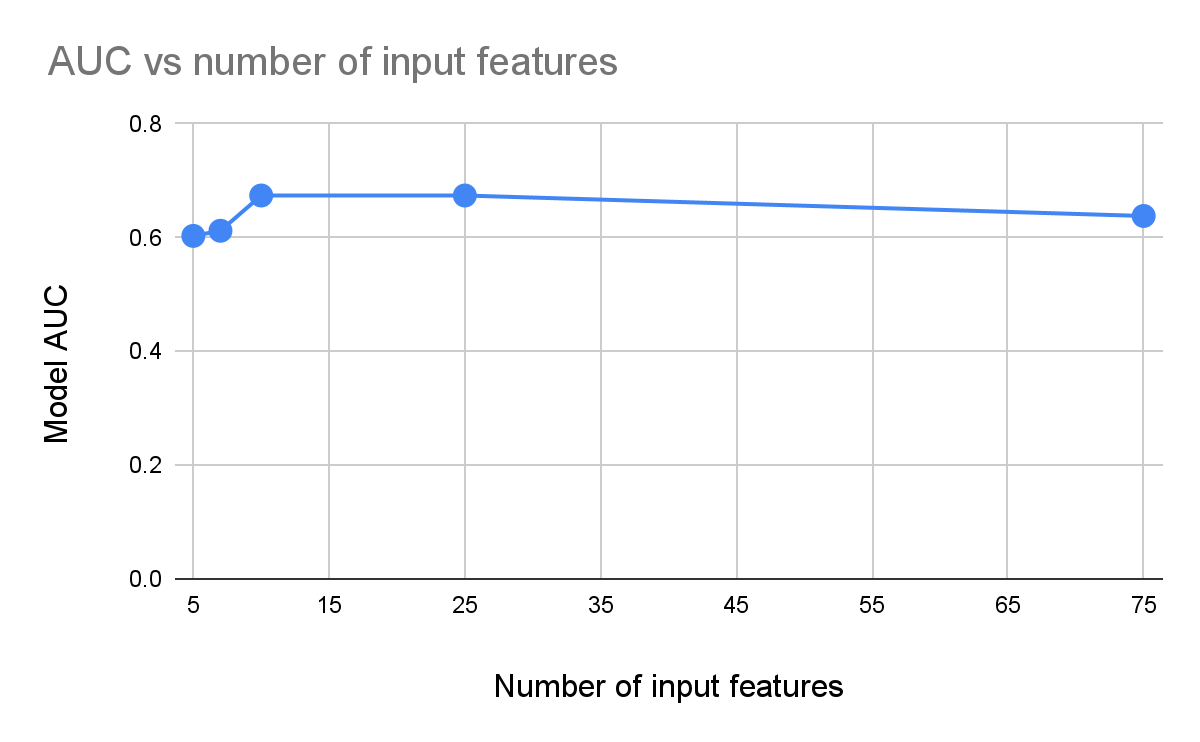


**Supplementary Figure 3: Variation in Area Under the Curve (AUC) by number of input features included in the model**
